# Effects of urban building structures on the distribution of breeding sites and ovitrap egg counts of *Aedes albopictus* in Pfaffengrund, Heidelberg, Germany

**DOI:** 10.1186/s13071-026-07655-z

**Published:** 2026-09-12

**Authors:** Charlie Wahl, Sven Lautenbach, Artin Tokatlian Rodríguez, Norbert Becker

**Affiliations:** 1 GIScience group, Institute of Geography, Heidelberg University, Im Neuenheimer Feld 368, 69120 Heidelberg, Germany; 2 HeiGIT gGmbH, Schloss-Wolfsbrunnenweg 33, 69118 Heidelberg, Germany; 3 Heidelberg Center for the Environment (HCE), Heidelberg University, Im Neuenheimer Feld 130.1, 69120 Heidelberg, Germany; 4 ICYBAC Mosquitocontrol GmbH, Georg-Peter-Süß-Str. 1, 67346 Speyer, Germany; 5 Centre for Organismal Studies, Faculty of Biosciences, Heidelberg University, Im Neuenheimer Feld 230, 69120 Heidelberg, Germany; 6 Institute of Dipterology (IfD), Georg-Peter-Süß-Str. 3, 67346 Speyer, Germany

**Keywords:** *Aedes albopictus*, Mosquito control, Urban structure, Germany

## Abstract

**Background:**

In recent years, the increased abundance and expansion of the invasive species *Aedes albopictus* has become a nuisance and health threat in urban areas of Germany. While intensive control and monitoring measures have shown promising results to reduce populations in urban areas, the growing challenges of these measures in newly affected areas have raised concerns regarding cost effectiveness and optimization in efficiency.

**Methods:**

We have used a suburban area (121 ha) with active control measurements in Heidelberg, Germany to study factors that influence the abundance of breeding sites and the number of eggs for *Aedes albopictus*. As part of control measurements, breeding sites at 1455 land parcels were recorded repeatedly between May and September 2023. In addition, egg counts in 39 ovitraps were recorded. We studied how strongly the effect of control measures on breeding sites was moderated by building type (single-family, terraced, multi-family house). In addition, we analyzed the importance of breeding site availability, proportions of parcels with different building type and the presence of uncontrolled parcels on *Aedes albopictus* egg laying activity in ovitraps within 100–300 m radius from the trap. Statistical analysis was based on generalized linear models and generalized linear mixed models. We fitted individual models for each buffer size and compared the model performance.

**Results:**

Terraced building plots contained the largest number of breeding sites and multi-family house plots the least. A higher share of terraced building plots in the surrounding area was associated with a higher maximum egg count in the ovitraps. In contrast, ovitrap maximum egg counts for *Aedes albopictus* were lower if the share of uncontrolled building plots in the buffer was higher and if more large breeding sites were present. The best model performance was observed for a buffer size of 200 m. If the temporal variability of the ovitrap egg count data was taken into account, no optimal buffer size could be derived and the effect of breeding site abundance became insignificant.

**Conclusions:**

Our results indicate that terraced house plots are positively associated with breeding site abundance and egg counts of *Aedes albopictus* in ovitraps. Control measures could be targeted on areas with this building type to increase the efficiency to reduce local breeding opportunities and consequently suppress local *Aedes albopictus* populations.

**Graphical Abstract:**

**Figure:**
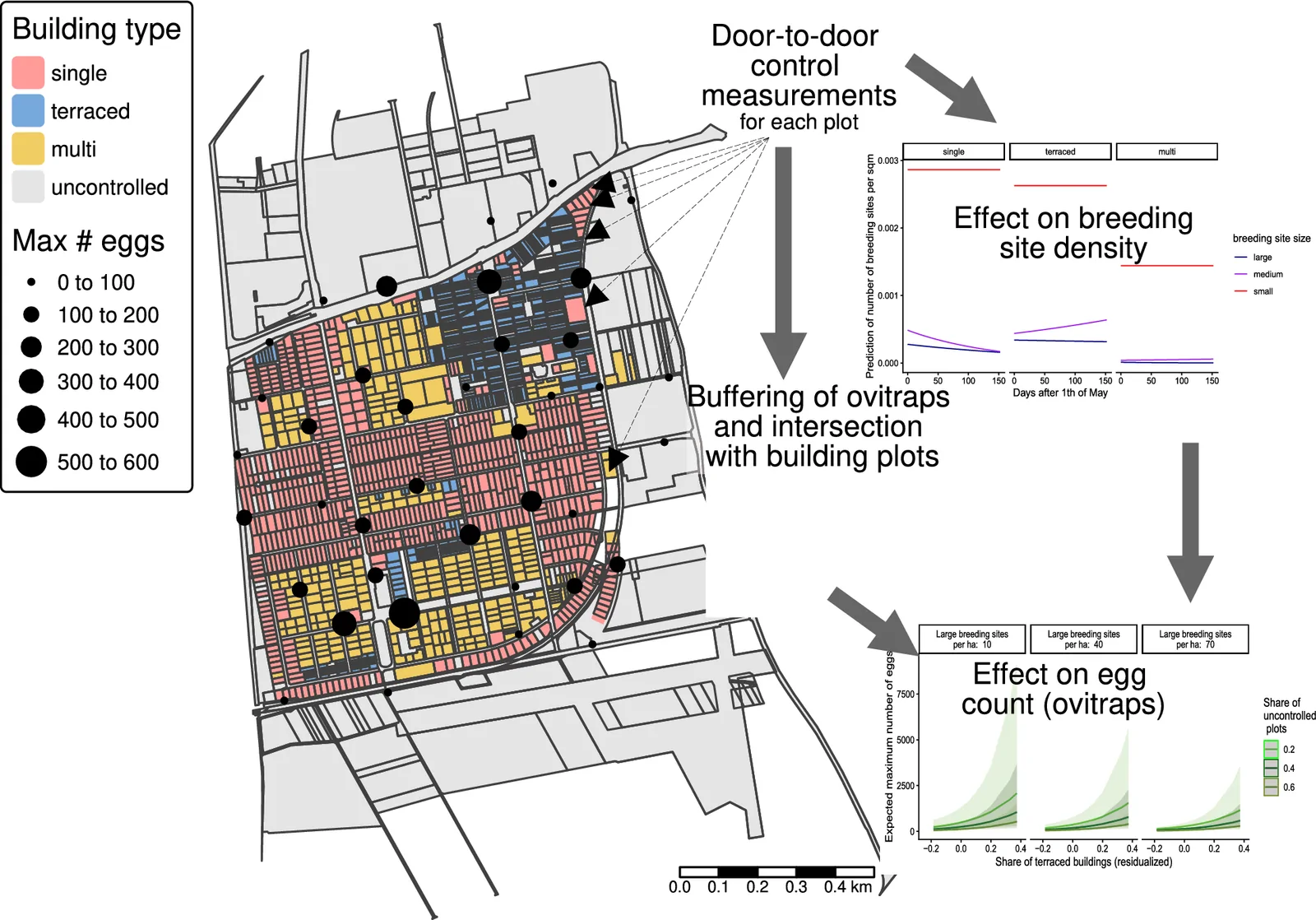

## Background

*Aedes albopictus* originates from tropical and subtropical Asia [1, 2]. Due to passive transport of eggs via “lucky-bamboo” plants and used vehicle tyres, the invasive species is now present on nearly all continents [1–3]. Known as an established vector for numerous diseases, such as dengue, Zika and chikungunya, *Aedes albopictus* poses an increasing potential public risk in more temperate zones [4–8]. In addition to its role as a disease vector, aggressive human-biting behavior of *Aedes albopictus* represents a major nuisance and reduces the quality of life in affected areas [9, 10].

The successful global distribution of *Aedes albopictus* is primarily supported by its adaptability: the eggs can survive extreme conditions including dryness and freezing temperatures, by entering a diapause until favourable conditions are encountered again [11–14]. This adaptability allows the species to thrive in diverse new habitats, ranging from natural ecosystems to urbanized areas [4, 15, 16]. The breeding sites of *Aedes albopictus* are diverse, spanning from natural sites such as tree holes and trunks to artificial water-holding containers, such as cups, pots, saucers or water barrels [17]. Urban environments are particularly supportive for breeding due to the high abundance of artificial containers, which are characterized by a low organic content and high temperatures [17]. Together with other factors—such as higher temperatures due to the urban heat island effect—this frequently leads to higher *Aedes albopictus* densities in urban or suburban environments [15].

In Europe, *Aedes albopictus* was introduced at least three times independently into Albania, Northern Italy and Central Italy [18]. The mosquito has steadily expanded its range, driven by climate change [19] and transportation networks [20, 21]. Northern and Central Italy have been identified as the main dispersal centers in Europe during the initial stages of invasion [18]. In Germany, the species was first documented alongside key transportation highways and railway lines, such as the highway E35 which connects German regions—via Switzerland—with Northern Italy, where *Aedes albopictus* has been already established since 2007 [20, 22, 23]. Over time, the number of documented overwintered populations in southern Germany has increased [24], highlighting its growing adaptability to the climatic conditions [25, 26]. As a result, the *Aedes albopictus* population in southern Germany has to be considered established [24]. For Heidelberg, an established population has been recorded since 2015 [27].

The ongoing *Aedes albopictus* populations in the Upper Rhine Valley, as indicated by regular monitoring and control efforts [26, 28], emphasize the importance for more effective strategies to regulate its distribution in Germany. We investigated this at the neighborhood of Pfaffengrund, Heidelberg. For this neighborhood recent observations highlighted hot spots of mass breeding sites in specific areas—underlying factors have, however, not been investigated so far [26, 28].

Control strategies for *Aedes albopictus* populations in Germany typically include an early detection and regular removal of potential breeding sites in combination with a cautious application of the protoxins of the bacterium *Bacillus thuringiensis* var. *israelensis* (Bti) as a control agent to water bodies [29, 30]. These measures are typically complemented by activities that involve the inhabitants. On private properties control measures have to face the additional burden to get the owner’s permission [10, 30]. Private parcels that remain uncontrolled can be assumed to act—together with other uncontrolled areas—as a reservoir for *Aedes albopictus*. To the best of our knowledge, the impact of uncontrolled areas in neighborhoods subjected to mosquito control measurement has not been quantified yet. Quantifying the effect of uncontrolled areas could increase awareness by property owners and subsequently enhance mosquito control effectiveness.

The door-to-door controls—which consists of a manual treatment of breeding sites by trained stuff—has been shown to be effective in a case study in Italy [31]. However, it remains labor and cost intensive [30, 32]. Door-to-door controls are frequently complemented by alternative control measures [33]. These include—among others—hotspot source reduction [34], sterile insect technique [30, 32, 35], the sterilization of wild female mosquitoes with its natural endosymbiont Wolbachia [36, 37], or the use of indigenous copepods [8, 38]. An important component of mosquito control is the information and involvement of the general public [33]. The integrated vector management approach implemented in the Ticino canton, Switzerland which focuses primarily on awareness-driven source reduction and larviciding, has shown to effectively control the *Aedes albopictus* population [23].

What is missing from our perspective is a deeper understanding to what degree aspects of urban morphology and building patterns affect the effectiveness of door-to-door campaigns. We aim to contribute closing this knowledge gap by an analysis performed in a suburban neighborhood in Heidelberg, Germany with an established *Aedes albopictus* population. The aim of this study is to analyze the effect of urban morphology on the availability of breeding sites and to investigate the influence of breeding site abundance and size on egg-laying activity. We hypothesized, that urban morphology—here represented as different building types—is associated with behavioral differences of inhabitants which might moderate breeding site availability and indirectly the abundance of *Aedes albopictus*.

## Methods 

### Study area

**Fig. 1:**
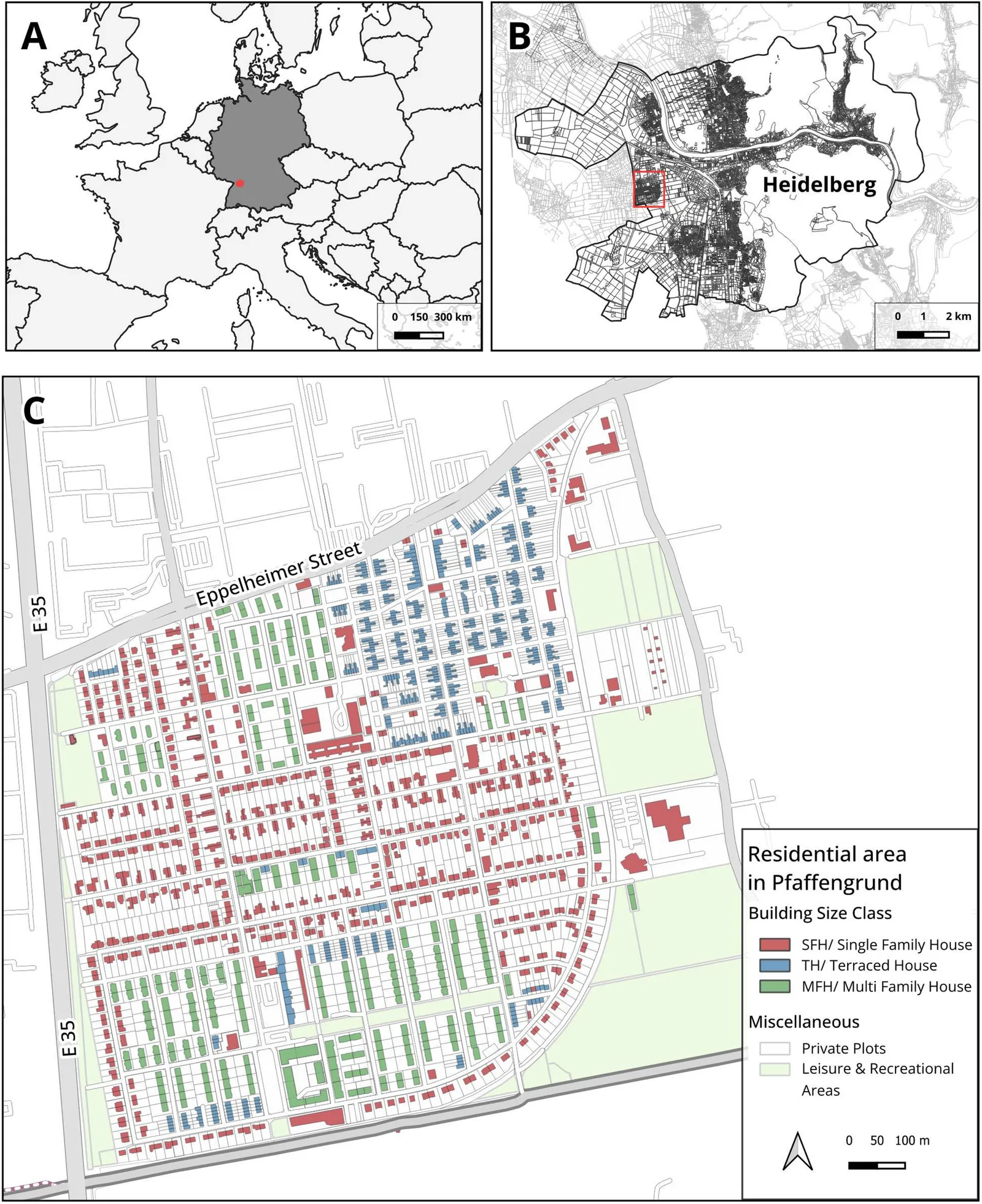
Overview of the study area in the neighborhood of Pfaffengrund in Heidelberg, Germany. **A** Position of Heidelberg in south–west Germany, Europe. **B** Location of the study areas in Pfaffengrund, which is located in the south–west of Heidelberg. **C** Residential areas in Pfaffengrund used in the case study. Single Family Houses (SFH) were primarily present in the center and surrounding areas, Terraced Houses (TH) mainly in the north–east and south and multi-family houses (MFH) dominated the southern part of the area, with few MFH in the north-western section. The area was surrounded by leisure and recreational areas. The highway E35 cut off the study area in the West. Datasource: LGL [41].

This study was carried out in the neighborhood of Pfaffengrund, located in the western part of Heidelberg with about 7500 inhabitants. Heidelberg with around 160,000 inhabitants is located in the state of Baden–Württemberg in Southwest Germany. The development of the neighborhood started off in the early to mid 1900s under the garden city movement. This specific garden city layout, provided most properties with a back yard or garden, which is used either privately, on a shared basis, or as common ground. These outdoor spaces typically hold artificial containers for the storage of water, which can act as potential breeding sites for *Aedes albopictus*. The neighborhood is marked by a highway (E35, cf. Fig. 1) to the west and by a city highway (Eppelheimer Street) to the north. It is bordered by an industrial zone in the north, agricultural areas in the south, as well as allotment gardens and a sports ground to the east. The residential neighborhood with a size of 121 ha has been subject to regular door-to-door control measures since 2019.

We focused on the period between mid May and the end of September 2023 to capture the presumably most favorable meteorological conditions for successful *Aedes albopictus* development [23, 39]. Monthly average temperatures at 2 m above ground ranged from 22.0°C in July to 16°C in September. Minimum temperatures remained above 10°C from June to August and above 6°C in May and September [40].

Involvement of the inhabitants into the control measurements has been seen as an important measure by the city administration. Information about *Aedes albopictus* and control measurements are provided in digital and analog form by the city administration. Forms of involvement included bulk mailing of information material, open councils, public postings, interviews and news articles in regional newspapers and radio broadcasts and information on websites [27]. These measures at city scale augment information provided by the door-to-door controls described below.

### Data and preprocessing

#### Building types

Building properties formed the spatial units of analysis. These were derived from the authoritative land survey register ALKIS (*Amtliche Liegenschaftskataster Informationssystem*) [41], which provides plot boundaries and building footprints together with the road network and other auxiliary information, such as land use.

**Fig. 2:**
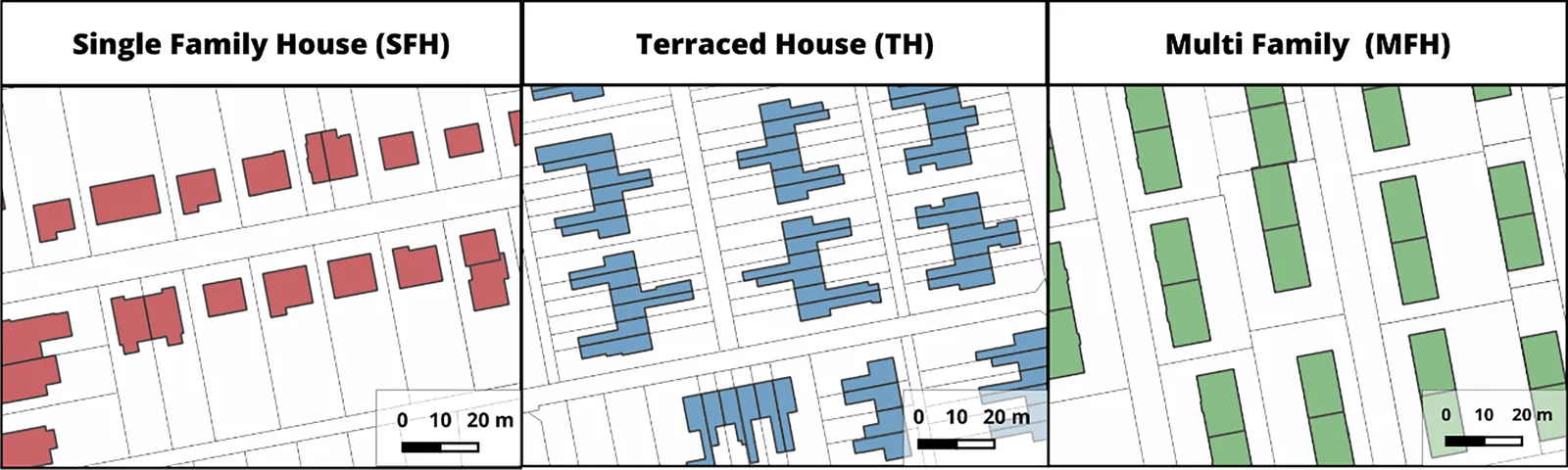
Comparison of the dimensions and shapes of the three present building size classes on private and semi-private plots of land: single-family houses (SFH) in red and terraced houses (TH) in blue on their associated private plots of land and multi-family houses (MFH) in green on their semi-private plots of land. Datasource: [41, 43].

To investigate how urban structures influence abundance of breeding sites of different size categories as well as ovitrap egg counts, we categorized each plot of land according to their building type class, based on the TABULA (*Typology Approach for Building Stock Energy Assessment*) project [42] and other typological studies [43]. Within our study area, we identified three dominant building types (cf. Fig. 2) in Pfaffengrund: single-family houses (*single*), terraced houses (*terraced*), and multi-family houses (*multi*) [44]. Single-family houses are defined by one or two story buildings in a single or semi-detached way, typically containing a single dwelling unit [43]. Individual buildings of these type are often surrounded by larger gardens and in a larger distance to the neighboring houses [43]. Terraced houses, also comprises one or two story buildings, typically designed to accommodate up to two households [43]. Buildings of this type are compactly arranged in continuous rows or minimal separation between neighboring buildings [43]. It is common to see a small front garden along the road and a back garden. Multi-family houses are defined as multi-story complexes comprising a greater number of apartments [43]. These buildings are typically situated alongside roads and lack privately utilized front or back gardens. Instead, residents utilize and share the green spaces as semi-private areas that can also be used by the public [43].

Outhousings and annexes were not considered. Plots of land primarily containing schools, churches or community centres were classified as residential buildings, as long as they received regular treatment during control measures.

#### Door-to-door controls

During door-to-door (DtD) controls all private plots of land in the neighborhood were visited by trained staff, who were supervised by the research team of ICYBAC GmbH. The first round of control in 2023 started in the first week of May. This relatively late start was motivated by the weather in 2023, as April was unusually cold with a couple of frost days [45]. Until end of September 2023 we conducted regular DtD control in two week intervals. Upon a visit to a household, each team asked for permission from the houseowner to examine the premises. If entrance to the property was granted, the entire outside premise was inspected for potential breeding sites such as containers potentially holding water. Within each premise we documented the presence of all containers capable of holding water which could work as breeding sites for *Aedes albopictus*. Depending on the potential water holding capacity the containers were categorized into predefined size classification of small, medium or large breeding sites (cf. Table 1). The potential breeding sites were not examined for presence of *Aedes albopictus* larvae.

The DtD visits were also used to inform and sensitize of the inhabitants: present inhabitants were informed about the risks and nuisances associated with *Aedes albopictus* and which control measurements could be used to reduce the *Aedes albopictus* population. In a next step, inhabitants were shown potential breeding sites on the plot of land. They were, furthermore, shown how to deal with the breeding sites and were encouraged to actively support the measures in between the control visits. The information procedure during the initial control visit took about 15 minutes. During follow-up control visits less time was spent on information, if not requested otherwise by the inhabitants. At the first follow-up visit at the beginning of the breeding season of each year, additional time is scheduled to refresh the knowledge of the inhabitants.

All potential breeding sites on the assessed properties were mapped into a GIS project and subsequently treated. Large containers were treated by Bti with predetermined dosages [30] and inhabitants were instructed how large containers could be inactivated by mesh nets to prevent large outbreaks and reduce the use of Bti treatments. Small to medium containers were emptied during control visits. If necessary, inhabitants were offered Bti tablets and instructed how to use them in between control visits. Properly inactivated breeding sites observed during the site visits were not counted as breeding sites.

Access was denied for several private plots of land (70 out of 1455), which were classified as uncontrolled areas for the subsequent analysis. In addition, all uninhabited plots of land, such as green areas and industrial sites, were also classified as uncontrolled areas for downstream analysis. For these sites, no control measures were implemented, with the exception of road drains, which were treated with Bti by the ICYBAC team at intervals of about 4 weeks. Roads were still treated as uncontrolled areas, as breeding sites such as garbage bins or grit containers were not treated. In addition, no information on breeding sites was collected for all contontrolled areas.

For the comparison across building types we calculated the number of breeding sites per hectare for each plot. As the number of control visits differed across the different plots (between 1 and 10 visits, q25=4, median=5, mean= 5.34, q75=7) we calculated the mean of breeding sites per plot across control visits. For plots that could not be accessed no information about breeding sites was available.

**Table 1:** Container size categorization by potential water holding capacity

Size category	Water holding capacity [liter]	Examples
Small	< 1	Small backyard water drains
		Parasol stands, flower pots, trivets
Medium	1–30	Water buckets, watering cans
		Lager water drains, cable ducts
Large	> 30	Rainwater barrels, water holding tanks

#### Ovitraps

The actual presence of *Aedes albopictus* was investigated using oviposition traps (ovitraps) from mid July to end of September 2023. A total of 39 ovitraps were distributed across the study area. The ovitraps could not be placed in a regular pattern as placement had to consider the local conditions such as access to the location and the possibility to properly place the ovitrap. The distance to the nearest trap was between 110.8 m and 250.5 m with an average distance of 149.5 m and the first and third quartiles of 124.8 m and 164.4 m. It was not always possible to inspect all ovitraps at the same date, e.g., if the owner or inhabitant was not present to grant access to the plot (cf. Table 2). The plots were revisited to inspect the plot—if this was not possible, information for this period was recorded as missing. Ovitraps consisted of a black plastic container (12 cm height and 12.5 cm diameter), each filled with one liter of tap water and a wooden stick, which serves as a substrate for egg-laying. An overfill protection 2 cm below the upper part of the container ensured that the ovitraps were not filled too much. The wooden sticks were removed, replaced and evaluated every two weeks. To compensate for loss by evaporation the ovitrap containers were filled up with fresh tap water during the control visits, if necessary.

**Table 2:** Dates at which ovitraps were inspected

Date	Number ovitraps inspected
2023-07-21	29
2023-07-26	16
2023-07-27	4
2023-08-10	7
2023-08-11	28
2023-08-24	39
2023-09-13	36

The morphology of the eggs was inspected visually to distinguish eggs by *Aedes albopictus* from those of *Aedes geniculatus* *Aedes koreicus* or *Aedes japonicus* [29]. For an additional quality control step, ovitrap sticks were allowed to hatch if the visual inspection was considered uncertain—which was the case in less than 10% of the inspections. The positive ovitrap sticks were then placed in individual plastic trays containing water and ground fish food for hatching. The fourth stage larvae were then identified under the stereo microscope [29]. The identification based on larvae and on the morphology of the eggs did not differ. As eggs from *Aedes koreicus* or *Aedes japonicus* cannot be safely distinguished on egg morphology, we cannot be sure to which of the two species these eggs belonged. From the eggs allowed to hatch none belonged to *Aedes koreicus*. At the time of writing, no occurrence of *Aedes koreicus* had been reported for Heidelberg. The number of eggs were entered together with the species information for each observation into the database and linked to the ovitrap location data.

#### Buffer calculation to characterize potential trapping ares of the ovitraps

**Fig. 3:**
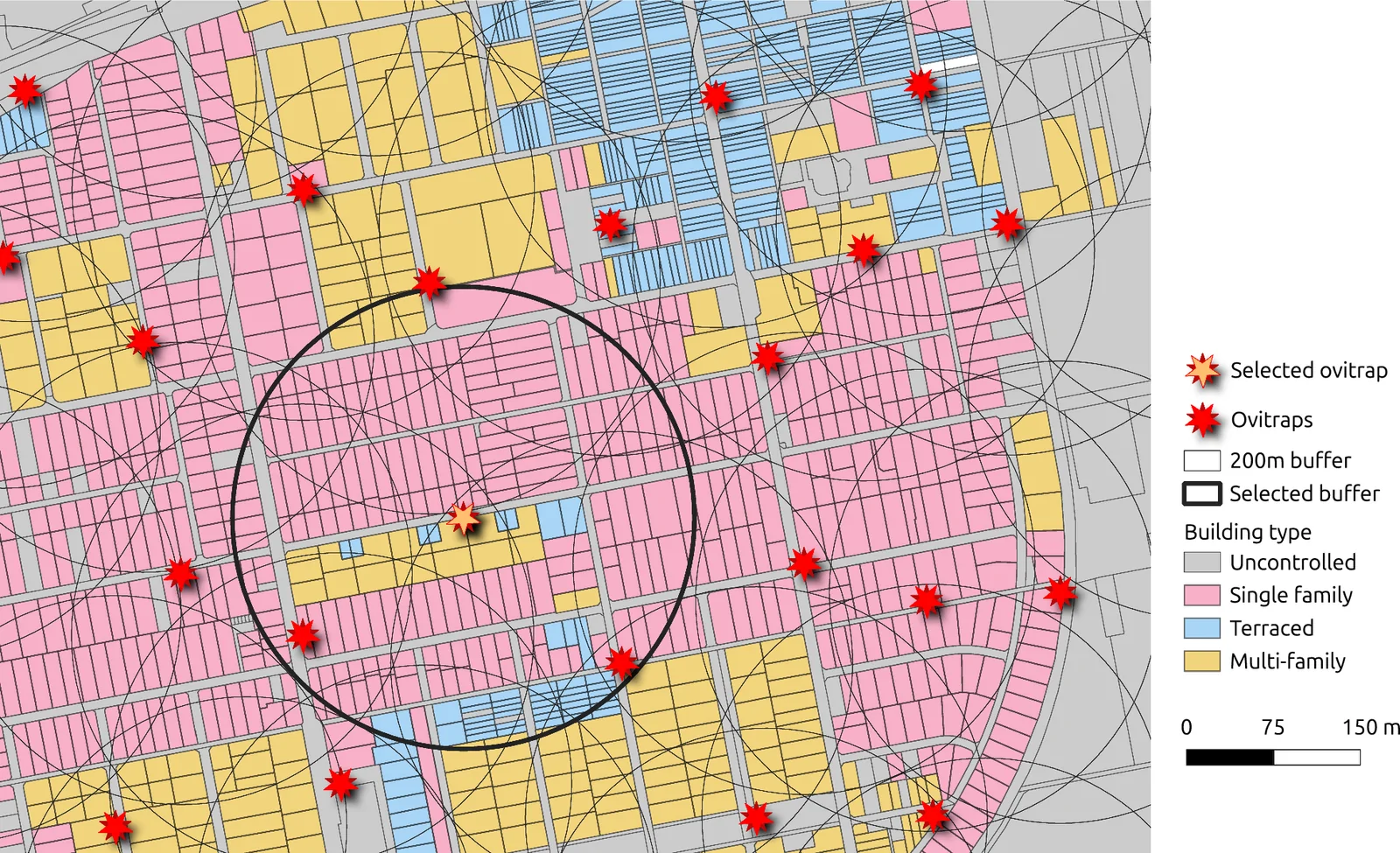
Buffer calculation. To relate information about ovitraps (egg counts) with information available for the building plots (building type, number of breeding sites), the ovitraps were first buffered. The buffers were then intersected with the building types. For each buffer, the share of each building plot inside of the buffer was calculated and then summarized by building type category. The breeding site information was already aggregated at the building plot level—i.e., no coordinates for the location of each breeding site were available. For the aggregation we assumed a homogeneous distribution of the breeding sites across the building plot. The resulting table was then joined with the ovitraps based on the ID of the ovitraps. To ease interpretation, one ovitrap is highlighted together with its 200 m buffer

To capture the influence of urban structure on the egg presence and abundance in ovitraps, we created buffers around the ovitraps and intersected the building parcels with the buffers (cf. Fig. 3). The choice of the buffer size should reflect the active dispersal range of *Aedes albopictus*—educated guesses could be based on flight range information. Previous studies in different areas affected by *Aedes albopictus* have assumed different flight distances for the species [27, 46–50]. For a nearby location ("Ochsenkopf") in Heidelberg a flight distance of 60–110 m had been identified. In light of these observations and the densely inhabited study area with high shares of private gardens, we hypothesized that a buffer size of 150 m around each ovitrap would be well-suited for the analysis. To test this assumption, we used circular buffers with five different sizes (100, 150, 200, 250, 300 m) around each ovitrap for our analysis.

The E35 motorway has high traffic volumes, which presumably acts as an artificial barrier for the dispersal of *Aedes albopictus*. Therefore, we decided to clip any buffer areas that exceeded the motorway. Assuming a homogeneous distribution of the breeding sites across the plot we calculated the area weighted sum of breeding sites per size category for each buffer. For each buffer we also calculated the share of plots of the three building types and uncontrolled plots—non residential areas or residential plots for whcih access was not granted.

### Statistical analysis

We used a count regression approach to study the association between two primary aspects: first, we checked for an effect of the control measures on the number of breeding sites by an analysis of the temporal trend. For plots with repeated visits we hypothesized a decline of breeding sites over time. This decline was assumed to be dependent on the building type and to differ between the different size classes. We used a generalized linear mixed model (GLMM) with a negative binomial distribution and plot size as an offset, the building plot as the grouping variable for a random intercept and an AR1 temporal autocorrelation structure. We used both the main effects as well as the interaction for date (days since first of May 2023) and building type as predictors in the model. The function glmmPQL from the MASS package was used to fit the GLMM. The selection of a negative binomial GLM was motivated by the overdispersion in the data [51, 52]. An alternative approach using a zero-inflated or a hurdle model [53] could be rejected as the data showed no sign of zero-inflation.

Second, we analyzed the impact of different plot characteristics on the number of eggs laid in the ovitraps. This analysis was done at the level of ovitraps using the calculated shares of building type classes, uncontrolled areas and breeding site metrics within the five circular buffer areas (100–300 m) surrounding the ovitraps. We used the maximum eggs count per ovitrap over the time period (mid May till end of September) as the response variable and tested effects of the number of breeding sites of different sizes and the share of the different building types in the buffer. We fitted separate generalized linear regression models for each of the five buffer sizes and compared the estimates and standard errors. As the number of breeding sites per plot changed over time, we decided to average the number of breeding sites per size class per plot across time.

As the number of breeding sites for the three size classes was associated with building type we residualized the effect of building type. Thereby, we controlled for the effect of correlation between the predictors and derived non-collinear predictors [cf. 54]. As only the sum of large breeding sites was associated with the number of eggs per ovitrap, we residualized only for the number of large breeding sites. More precisely, we used the residuals of linear models with the share of building type as the response and the area weighted sum of the large breeding sites as the predictor. The residualized predictors represent the effect of presence of the building type in the buffer after taken the effect of the number of large breeding sites into account. Residualization was done separately for each of the five buffer sizes tested.

We checked for spatial autocorrelation of the regression residuals of each of the three models by means of global Moran’s I [55], using a permutation test with 9999 permutations. Local pattern were analyzed by local Moran’s I [56], using the approach by Holm [57] to adjust for multiple testing. We have used several neighborhood definitions for robustness of the analysis: we used search distances of 300 and 400 m—which correspond to buffer sizes of 150 and 200 m—to define the neighborhood. Afterward, we adjusted weights in the spatial weight matrix by distance between the neighbors using an inverse distance weighted approach with the power parameter 1 and 2. Afterward, we row-standardized the spatial weight matrix.

To consider temporal variability, we additionally used a GLMM to model the egg count over time. This allowed us to incorporate the temporal variability of abundance of the breeding sites: as the inspection of the ovitraps was performed at different dates than the control measurements, we used the number of breeding sites of the different size categories from the closest DtD visit available. We used the ovitrap ID as the grouping variable for a random intercept and an AR1 temporal autocorrelation structure. As the estimation of the θ term of the negative binomial distribution was unreliable in the context of the GLMM, we used a quasipoisson distribution instead. We tested the same predictors as for the GLM but added date as a orthogonal polynomial term of second order to reflect the temporal development. Similar to the GLM, we fitted the GLMM for each of the buffer sizes separately. Model fit was compared based on the marginal coefficient of determination for GLMMs [58] as the model structure was always the same.

Analysis was performed in R [59] using the following packages: ggplot2 (plotting, doi: https://doi.org/10.32614/CRAN.package.ggplot2) [60], spdep (spatial analysis, doi: 10.32614/CRAN.package.spdep [61], sf (geoprocessing, doi: https://doi.org/10.32614/CRAN.package.sf) [61, 62], tmap (thematic cartography, doi:10.32614/CRAN.package.tmap) [63], MASS (GLMM, doi: https://doi.org/10.32614/CRAN.package.MASS) [64], MuMIn [65] (marginal coefficient of determination, doi: https://doi.org/10.32614/CRAN.package.MuMIn), arm (plotting, doi: https://doi.org/10.32614/CRAN.package.arm) [66] and dplyr (data wrangling, doi: https://doi.org/10.32614/CRAN.package.dplyr) [67].

## Results

### Analysis of breeding sites

A total number of 1454 plots of land were controlled and documented, for 70 additional plots access was denied by the owner. The majority of the plots (48%) were classified as single-family houses, 34% as terraced houses and 18% a multi-family houses (cf. Table 3).

**Table 3:** Number of plots and number of breeding sites per ha—averaged across number of control visits—across building type. Breeding sites could only be counted at controlled building plots.

Building type	# Plots		Breeding sites per ha		
	contr.	uncontr.	Small	Medium	Large
Single family	689	30	46.9	11.0	16.5
Terraced	449	40	51.4	19.9	21.3
Multi-family	316	0	18.9	3.4	0.7

During the time period studied, around 21,688 breeding sites were documented for all visited plots: 4,581 large, 3,705 medium and 13,402 small breeding sites. The documented number of breeding sites normalized by the number of visits and by plot area differed by size category of the breeding site and across the building types (cf. Table 3). The number of breeding sites per hectare observed was lowest for multi-family houses. Terraced and single-family houses were more similar with respect to the density of breeding sites. Still, terraced house plots showed consistently higher densities than single-family houses. The densities for small breeding sites were higher than the other two size categories. For plots with single-family and terraced houses, the density of large breeding sites was higher than for medium breeding sites, while the opposite was observed for multi-family plots.

**Fig. 4:**
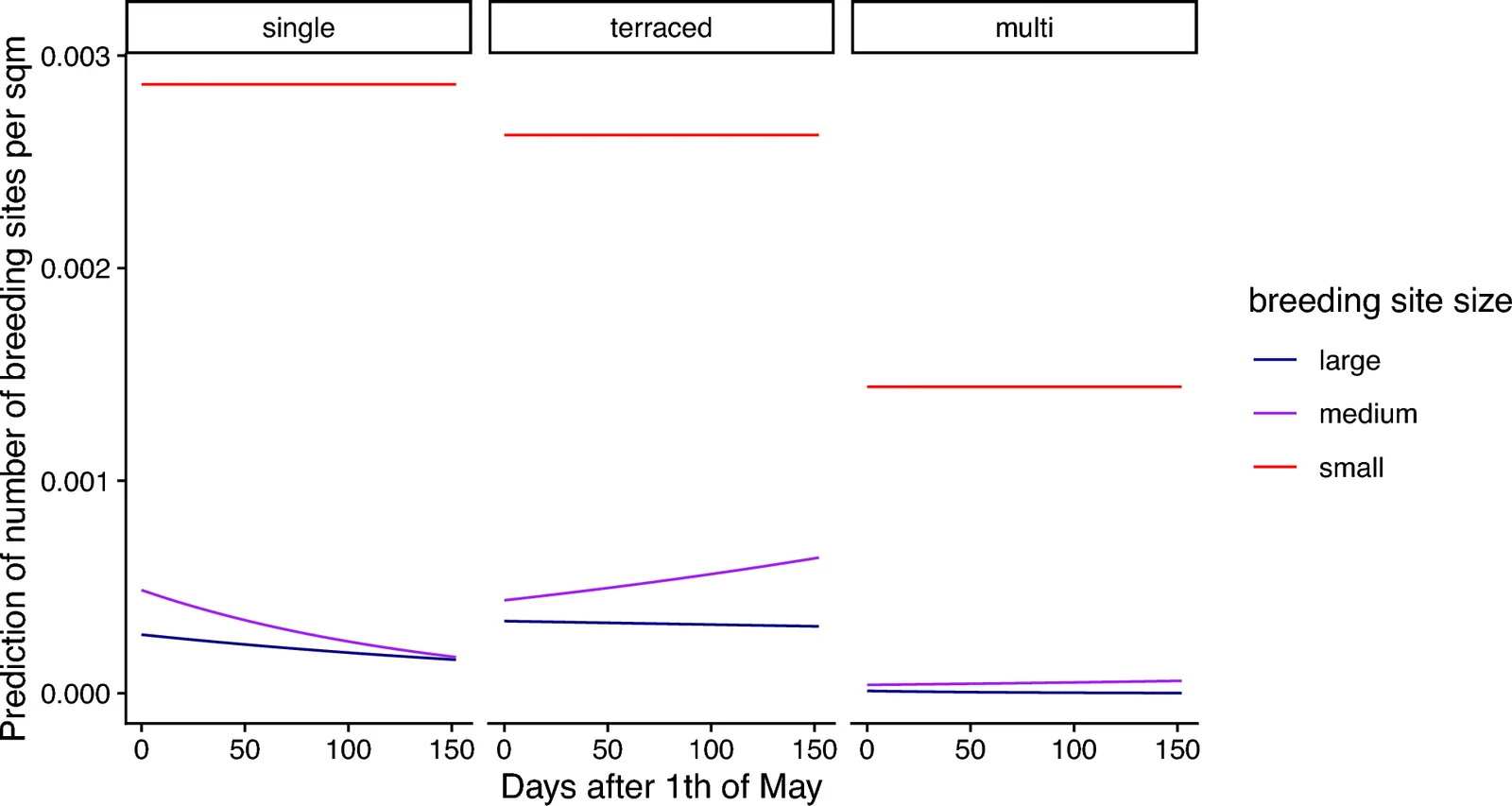
Prediction plots based on the GLMM for the number of breeding sites per square meter depending on the date (in days after 1th of May) and the building type of the parcel. The colors differentiate between the three size categories of the breeding sites.

The regression models (cf. Table 4) indicated clear differences between breeding site densities for multi-family and single-family houses for breeding sites of all three size categories. The breeding site density for terraced buildings did not differ significantly from single-family houses. On average, the density for large breeding sites declined over time—i.e., with increasing number of control visits (cf. Fig. 4). For medium breeding sites the effect was mixed: the estimated effect indicated a decrease of medium breeding site density for single-family houses. However, for terraced houses and multi-family houses, the regression coefficients indicated an increase over time. For small breeding sites, the effect of time was insignificant. The variance between the building plots was high as indicated by the random intercept variance component ($\sigma _{Intercept}$).

**Table 4:** Regression model results for the number of breeding sites of different sizes.

Predictor	Value	Std.Error	*DF*	*t* value	*P* value
Large breeding sites					
Intercept	− 8.194	0.1360	5823	− 60.252	$<10^{-16}$
Date	− 0.004	0.0003	5823	− 11.000	$< 10^{-16}$
Building type terraced	0.208	0.2238	1423	0.928	0.354
Building type multi-family	− 3.019	0.3251	1423	− 9.812	$< 10^{-16}$
Date: building type terraced	0.003	0.0006	5823	4.978	$6.6 \cdot 10^{-07}$
Date: building type multi-family	− 0.010	0.0016	5823	− 6.140	$8.8 \cdot 10^{-10}$
$\sigma _{Intercept}$	3.272				
$\sigma _{Residual}$	0.366				
$\phi _{AR1}$	0.501				
Medium breeding sites					
Intercept	− 7.630	0.0976	5823	− 78.181	$< 10^{-16}$
Date	− 0.007	0.0005	5823	− 12.679	$< 10^{-16}$
Building type terraced	− 0.104	0.1664	1423	0.166	0.532
Building type multi-family	− 2.503	0.2033	1423	− 12.311	$< 10^{-16}$
Date: building type terraced	0.009	0.0010	5823	9.334	$< 10^{-16}$
Date: building type multi-family	0.010	0.0013	5823	7.217	$5.9 \cdot 10^{-13}$
$\sigma _{Intercept}$	2.154				
$\sigma _{Residual}$	0.456				
$\phi _{AR1}$	− 0.101				
Small breeding sites					
Intercept	− 5.855	0.0504	5826	− 116.153	$<10^{-16}$
Building type terraced	− 0.087	0.0839	1423	− 1.034	0.301
Building type multi-family	− 0.687	0.0914	1423	− 7.509	$1.0 \cdot 10^{-13}$
$\sigma _{Intercept}$	1.248				
$\sigma _{Residual}$	0.634				
$\phi _{AR1}$	− 0.008				

Regression coefficients and standard errors are reported at the link scale. Date was coded as the number of days since the first of May 2023. The building type-related coefficients are shown using treatment coding, i.e., the coefficients show the difference compared to single house plots. A negative binomial GLMM with log link, random intercept and AR1 correlation structure was used was used.

### Analysis of maximum egg count in the ovitraps

**Fig. 5:**
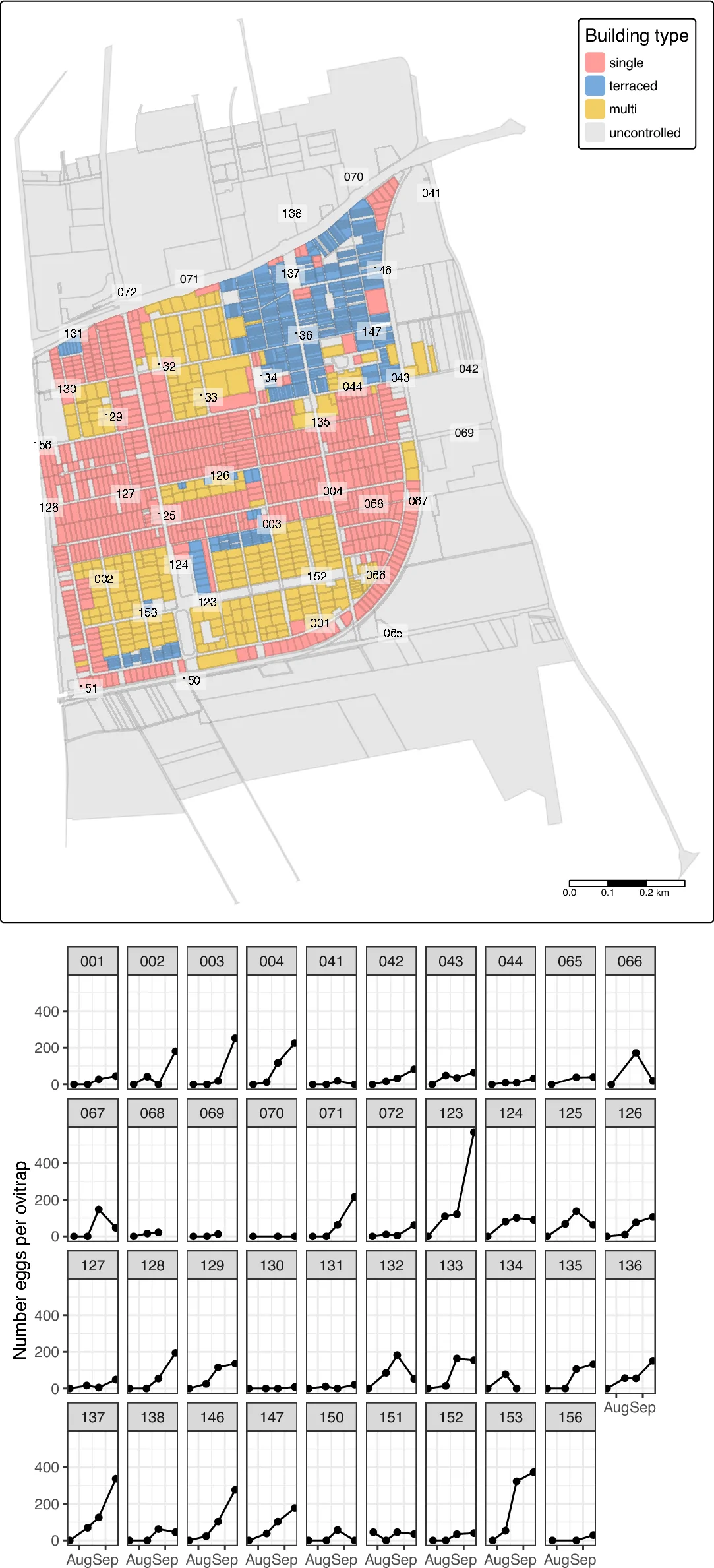
Number of eggs per ovitrap over time and map of the correponding locations. The ID of the ovitrap is displayed in the map and in the panel of each times series plot. The time shown is the time when the trap was collected at the parcel.

We documented 7697 eggs of *Aedes albopictus* in 39 ovitraps from 15th of June to the 19th of September. The mean egg count per ovitrap was 66.8 eggs. The highest eggs count per trap was 569 eggs. The different sites showed different temporal patterns (cf. Fig 5): about half of the sites showed no clear trend over time, while the rest showed a peak at the beginning or at mid of September.

While *Aedes japonicus* has been frequently observed in Heidelberg, eggs of this species—or of *Aedes koreicus*—were rarely encountered in the ovitraps within the study region. Eggs of *Aedes japonicus*—or *Aedes koreicus*—were present at five different ovitraps. At four ovitraps *Aedes japonicus*—or *Aedes koreicus*—eggs were present in May, then no *Aedes albopictus* eggs there present. These four sites were located in the north east of the study region, three of them neighboring an allotment site. At the fifth ovitrap 10 eggs were present in July, while 121 eggs of *Aedes albopictus* were present. The site was located in the south of the study region, about 150–200 m away from an allotment site. We hypothesized, that the absence of *Aedes japonicus* within the study area for most of the time was be related to the large distance (shortest distance about 3 km) of Pfaffengrund to forested areas. This would be in line with results from other parts of Germany [68] and Japan [69].

**Fig. 6:**
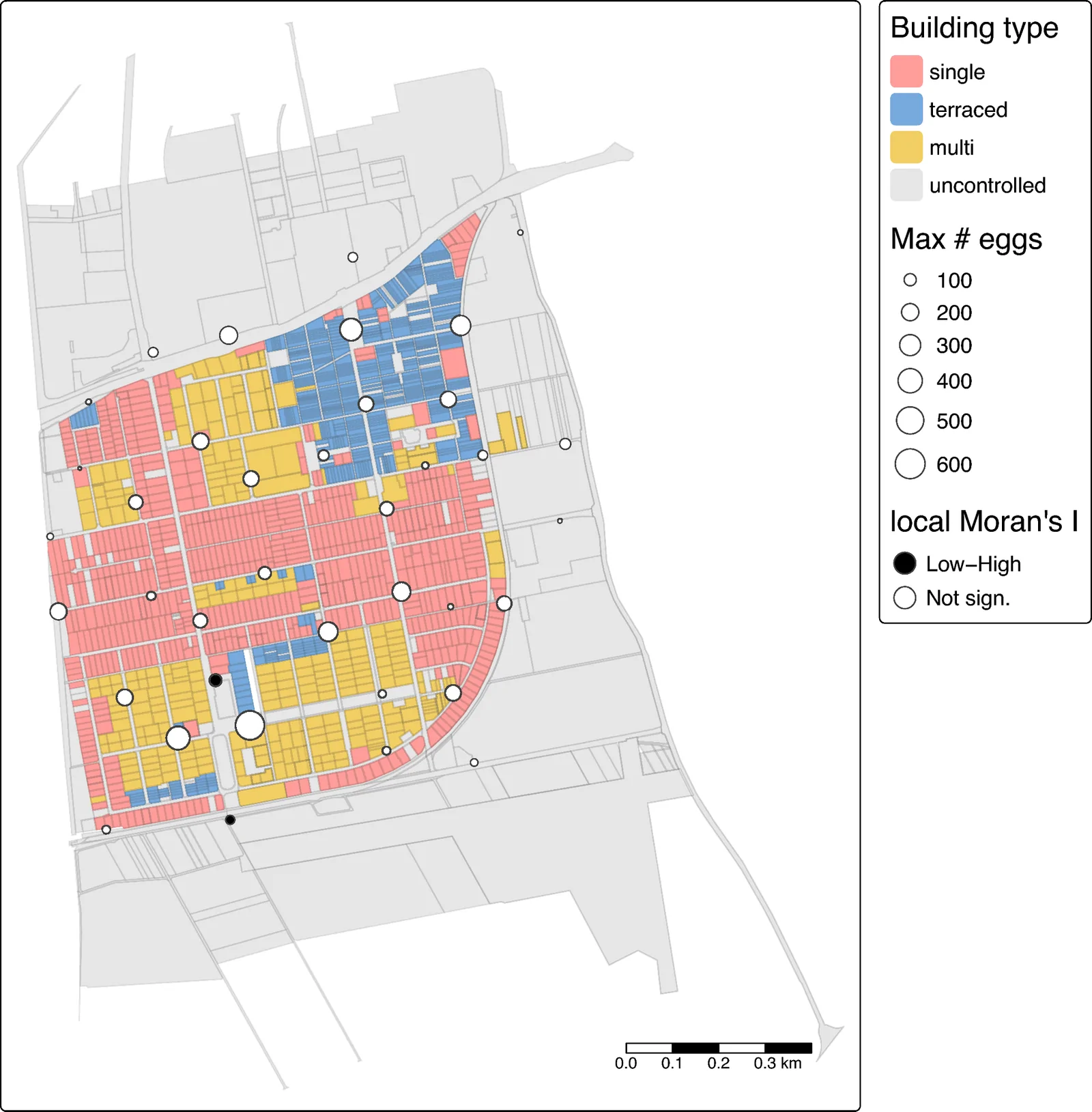
Position and maximum number of eggs per ovitrap. Local Moran’s I was used to identify significant spatial pattern. Two locational outliers (low–high, low values in a neighborhood of high values) were identified. The plot was based on a 300 m neighborhood definition together with an inverse distance weighted scaling (power=2) of the spatial weight matrix, followed by a row standardization—the other neighborhood definitions returned the same pattern. The uncontrolled areas consist of industrial areas in the north, sport sites and allotment gardens in the east as well as agricultural fields in the south of the study area. In addition, parcels where access was not granted belong to the uncontrolled parcels as well.

The spatial distribution of the maximum number of eggs per ovitap showed high variability across space without an overly clear pattern (cf. Fig. 6). Ovitraps outside of the populated area showed low egg counts. Global Moran’s I indicated no significant spatial pattern across all tested specifications of the spatial weight matrix (*P* value always above 0.18). Local Moran’s I identified two points in the southern region dominated by multi-family houses as interesting: two ovitraps with lower maximum egg counts than expected (locational outliers, low–high).

The regression models (cf. Fig. 8) showed—in accordance with our expectation—that the maximum number of eggs per ovitrap was lower the higher the share of uncontrolled areas in the buffer. This negative effect of a higher share of uncontrolled areas could be explained by the higher number of larger breeding sites available at inhabited but uncontrolled parcels. The direction and order of magnitude was similar for all five buffer sizes. The *P* value decreased with increasing buffer size up to 200 m (*P* values 0.00121, 0.00018 and $7.23 \cdot 10^{-5}$ for 100 m, 150 m and 200 m, respectively). For larger buffer sizes, the *P* values increased (*P* value = 0.00372 at 250 m) and became marginally significant (*P* value = 0.0551) at 300 m buffer size.

For all buffer sizes we found that a higher number of large breeding sites within the buffer was associated with a lower maximum number of eggs per ovitrap. The direction and order of magnitude was similar for all five buffer sizes, the coefficient estimate was not significant for 100 m and marginally significant for 150 m (*P* value = 0.093) and 200 m (*P* value = 0.053)—at 250 m and 300 m the coefficient was insignificant (*P* value = 0.239 and *P* value= 0.456 at 250 m and 300 m, respectively). This marginally significant effect—at 150 m and 200 m buffer sizes—could as well be explained by the larger amount of alternative breeding sites that made the ovitraps less attractive for egg laying.

**Fig. 7:**
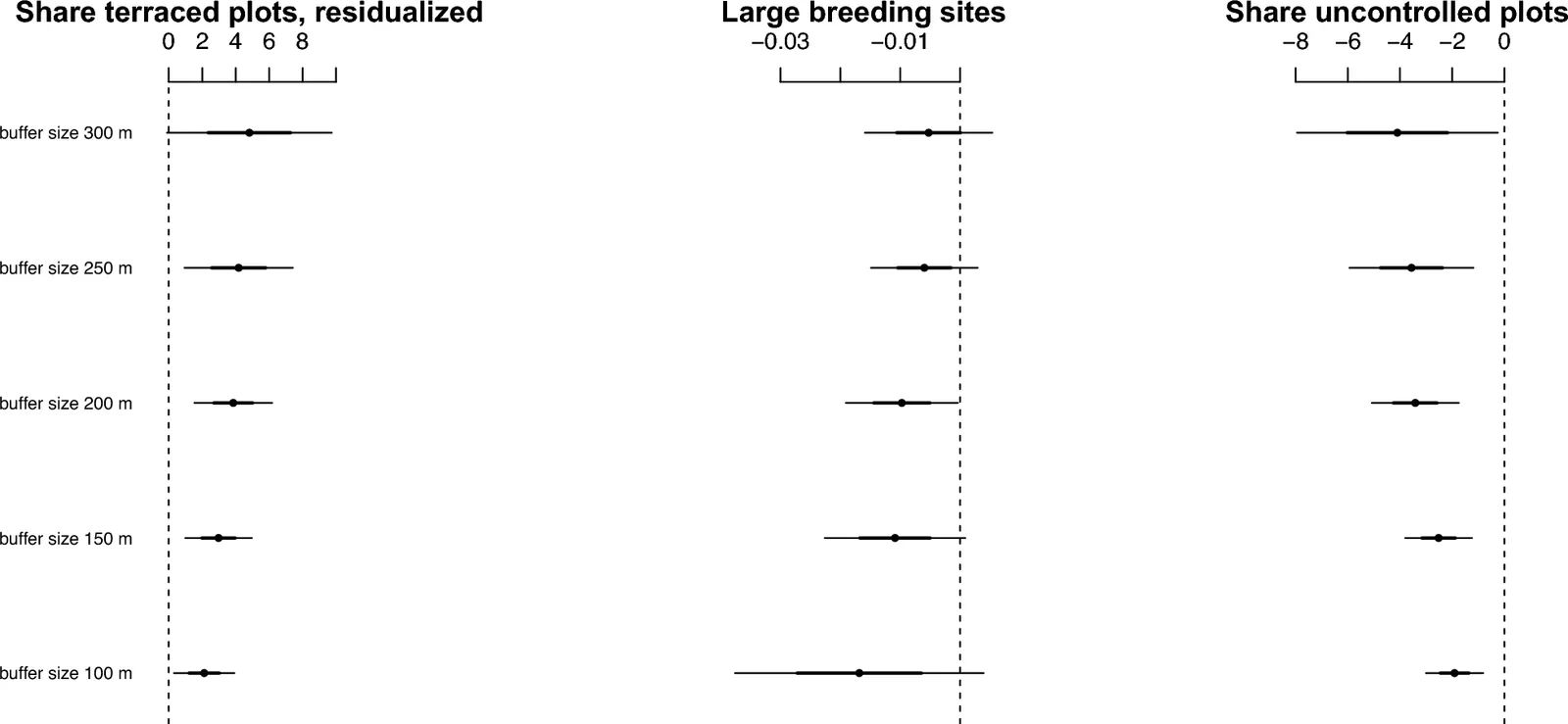
Coefficient plots of the regression models (GLM) for the maximum number of eggs per ovitrap. The plots allow a comparison of the coefficients of the five models for varying buffer sizes around the ovitraps. The dots represent the coefficient estimates, the thick line the standard error of the estimation and the thin line the 95% confidence interval. The dashed vertical lines indicate zero. Results are shown at the link scale.

After controlling for the effect of large breeding sites, the residualized share of terraced buildings was associated with a higher maximum number of eggs per ovitrap. Therefore, independent of the availability of breeding sites in the surrounding, ovitraps placed in areas dominated by terraced buildings were more attractive for egg laying by *Aedes albopictus*. The direction and order of magnitude was similar for all five buffer sizes, the coefficient size increasing with the buffer size. The *P* value of the coefficient estimate decreased strongly with buffer size up to 200 m: the *P* value dropped from 0.02 at 100 m buffer size to 0.003 at 150 m and further to 0.0008 at 200 m. At larger distances the *P* value increased (*P* value = 0.012 at 250 m and *P* value = 0.076 at 300 m buffer size) (Fig. 7).

The models were, however, only capable of explaining between 21% and 29% of the deviance in the response. Explained deviance first increased with increasing buffer size up to a buffer size of about 200 m before decreasing again (cf. Fig. 9).

**Fig. 8:**
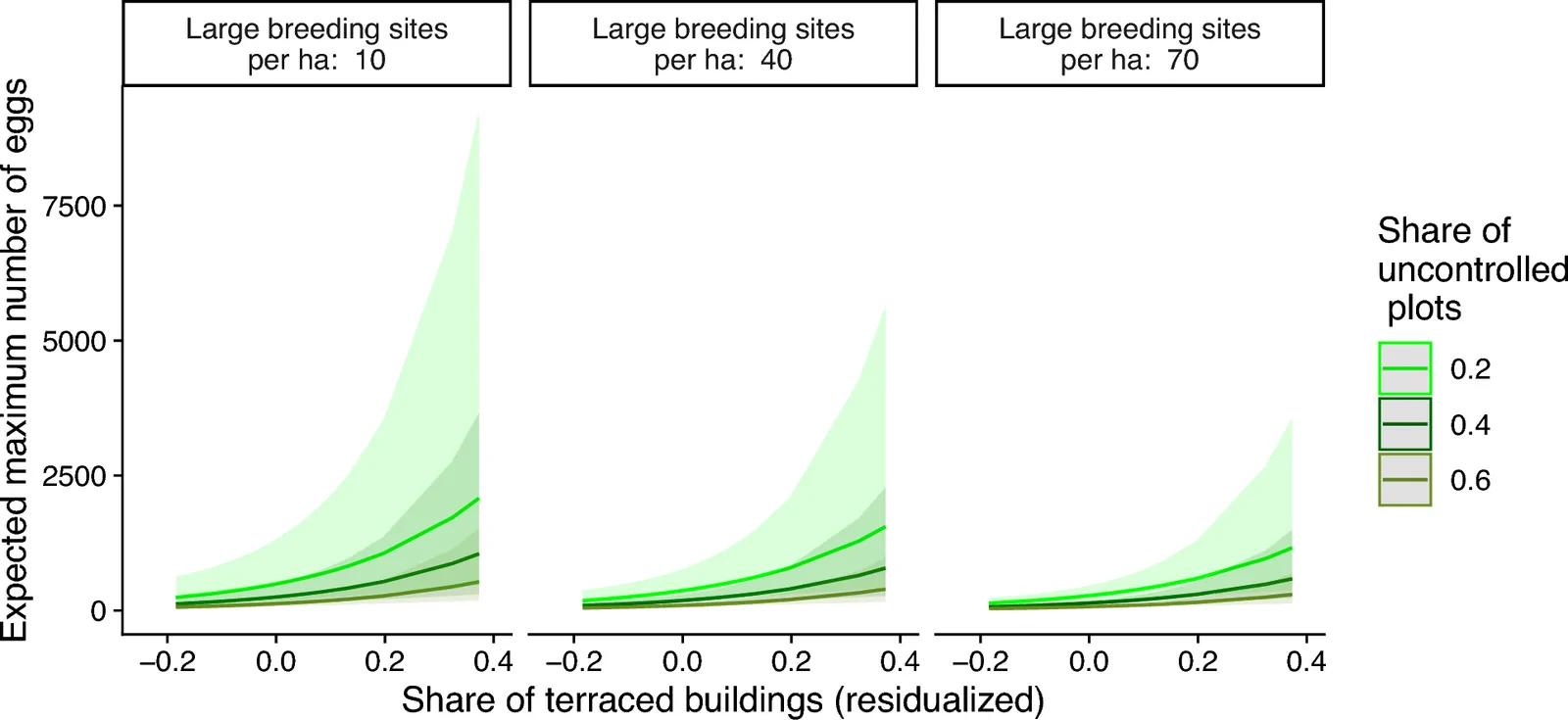
Effect plot of the regression model for the maximum number of eggs per ovitrap. The model for the 200 m buffer size is shown. Dashed lines represent the 95%-confidence band.

**Fig. 9:**
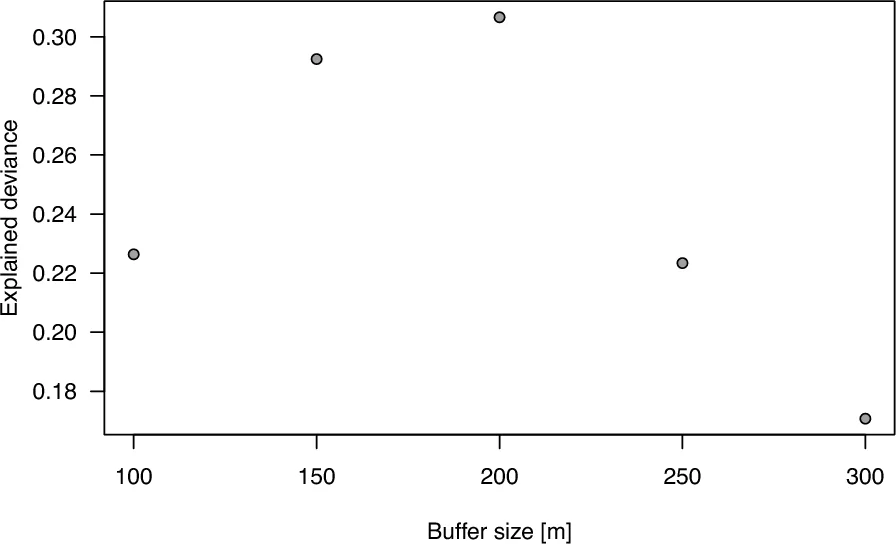
Explained deviance of the regression models (GLM) for the maximum number of eggs per ovitrap. The plots allow a comparison of the coefficients of the five models for varying buffer sizes around the ovitraps. As the models always used the same number of predictors the explained deviance can be used to compare the goodness of fit directly.

**Fig. 10:**
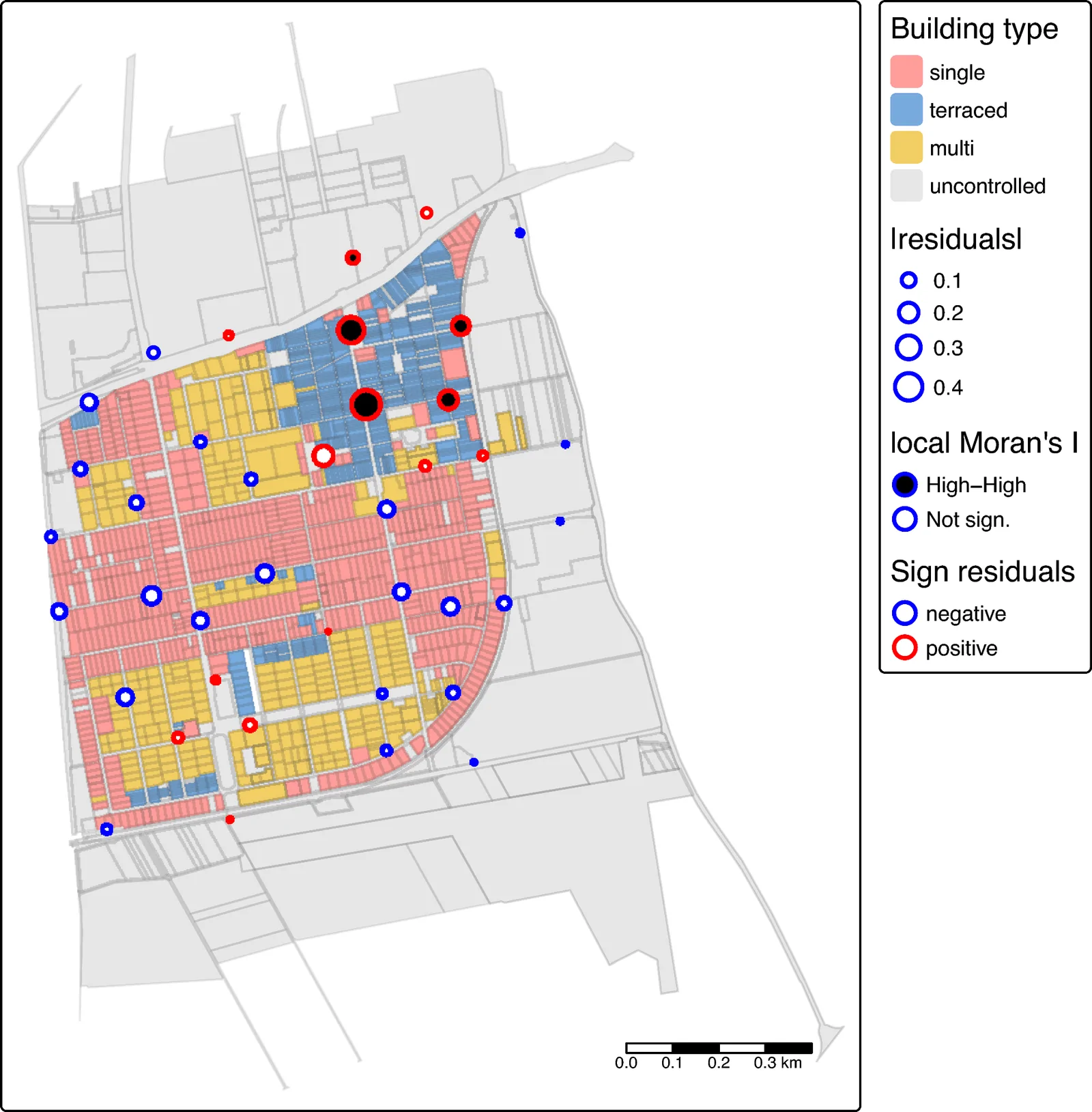
Residuals of the regression model (150 m buffer size) for the maximum number of eggs per ovitrap. Local Moran’s I was used to identify significant spatial pattern. The plot was based on a 300 m neighborhood definition together with an inverse distance weighted scaling (power = 2) of the spatial weight matrix, followed by a row standardization—the other neighborhood definitions returned the same pattern. One cluster of high residuals can be spotted around the terraced building type cluster in the north–east.

The residuals showed no global autocorrelation according to global Moran’s I (*P* value above 0.5 for all four spatial weigh matrices used). Local Moran’s I identified a cluster of five ovitraps with high number of eggs in the large terraced area in the north–east (cf. Fig. 10). The rest of the ovitraps showed no significant spatial structure according to local Moran’s I.

### Analysis of ovitrap egg counts over time

**Fig. 11:**
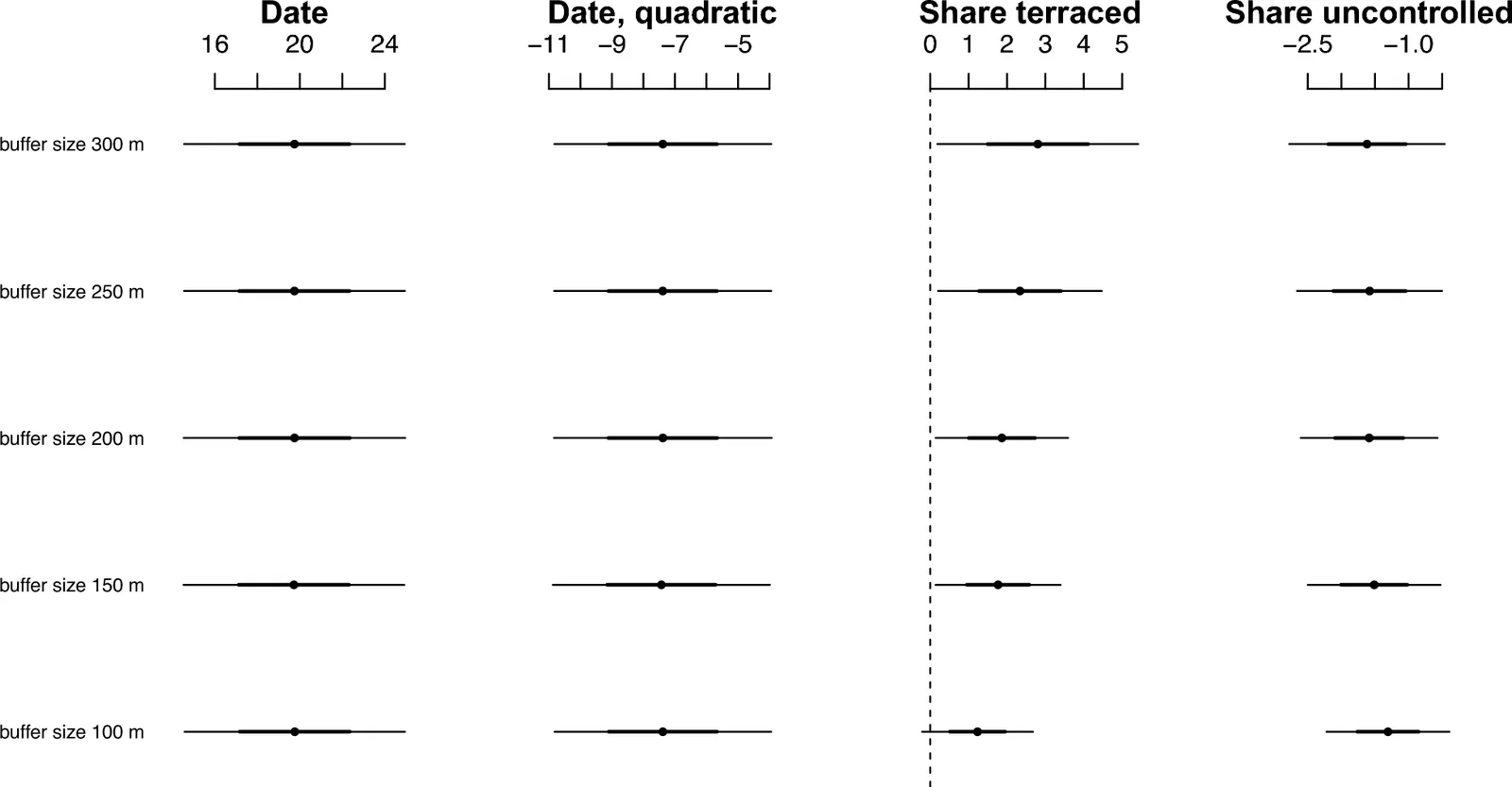
Coefficient plots of the GLMMs for the number of eggs per ovitrap for the different inspection dates. The plots allow a comparison of the coefficients of the five models for varying buffer sizes around the ovitraps. The dots represent the coefficient estimates, the thick line the standard error of the estimation and the thin line the 95% confidence interval. The dashed vertical lines indicate zero. Results are shown at the link scale.

For the analysis of the egg counts over time, the residualized regression coefficients for the number of breeding sites were clearly not significant. As the temporal autocorrelation coefficient was not significantly different from zero, we removed that structure from the GLMM. The model results were—not unexpectedly—dominated by the temporal development (predictor *date*) as indicated by partial pseudo-$R^2$. The model performance—measures by the pseudo-$R^2$ did not reveal much variation for model with different buffer sizes (marginal coefficient of determination between 0.74 and 0.75 and the conditional coefficient of determination around 0.82). The regression coefficient estimates for the linear and quadratic effect of *date* did not differ across the models for the different buffer sizes (cf. Fig. 11). The regression coefficient estimate for the share of terraced buildings showed always a positive association with the egg count—the size was about half of the regression coefficients for the GLMs used for the aggregated egg counts (cf. Fig. 7). The estimates coefficients were larger for larger buffer sizes and showed a similar pattern than for the GLMs used for the aggregated egg counts. For the share of uncontrolled plots the model estimated a significant negative effect which was about half the size of the coefficients estimated for the GLMs used for the aggregated egg counts. The coefficient estimate grew from 100 m to 200 m buffer size and stayed more or less stable for larger distances (cf. Fig. 11), again showing a similar pattern as the GLMs used for the aggregated egg counts (cf. Fig. 7). Using a simpler model with only a linear term for *date* did lead to a strong decline of the pseudo-$R^2$ values ($R^2_{marginal}$ dropped from 0.75 to 0.51 and $R^2_{conditional}$ dropped from 0.82 to 0.65) but revealed almost identical coefficient and standard error estimates for the other predictors.

## Discussion

Previous studies have primarily examined open areas in private properties when assessing breeding sites abundance and ovitrap sampling [7, 30, 70]. This study extends this analysis by adding building type classes to the analysis. This perspective helps to distinguish urban structures that may provide more suitable breeding habitat opportunities than others.

### Factors effecting breeding site abundance

We assumed that the management of associated gardens and other open spaces differed between the three classes, potentially making some plots more attractive to *Aedes albopictus*. While the control measurements are initiated by the members of the ICYBAC company, support by the inhabitants is beneficial for the success of the measures. Inhabitants were informed to empty containers filled with water or to cover containers and to apply Bti autonomously. The level at which these responsibilities are accepted by the inhabitants might differ depending on the building type. For multi-family houses, responsibility for—commonly used—open areas could be lower than for the other two classes. Containers owned by others might not be approached or it might be assumed that a necessary action was already undertaken by other inhabitants. On the other hand, facility managers might be more efficient in controlling breeding sites. For single-family houses the open space area can be assumed much bigger than for terraced houses, which might lead to a less consistent implementation of supporting activities than for the terraced houses. In addition, socio-economic factors, that can be expected to differ between the three building types, might affect how the gardens are managed. As fine-grained socio-economic data are hard to get in the German context, building type information might provide an easily accessible proxy.

The examination of the potential breeding sites on private properties revealed that—unexpectedly—terraced house plots consistently held the highest density of breeding sites across all size categories—closely followed by single-housing plots (cf. Table 4). Multi-family plots showed a much lower density. This might be caused by the differences in plot size as the multi-family plot tend to be bigger than terraced or single-family house plots. Inhabitants might place objects that represent potential breeding sites only on small parts of the shared ground (presumably close to their doors), leaving large parts without man-made/artificial breeding sites and, therefore, unsuitable for *Aedes albopictus*. In comparison, single-family and terraced house plots allow more privacy and individualized use of open areas. This could promote residents to place a greater variety of containers and, therefore, higher breeding site counts in their open areas. Terraced building areas have small outside areas, so the density of objects might be higher. Another aspect could be, that it might be sufficient if one of the inhabitants of a multi-family house or the facility manager gets sensitized for the importance of the control for breeding sites to keep the number of breeding sites—in commonly used areas—low. Shared outdoor space might, therefore, be easier to turn into a less suitable environment for *Aedes albopictus*. Nevertheless, it is important to also consider private facilities—e.g., balconies—as possible unreachable areas in control measures. Future research on differences in resident behavior and garden usage across different building types is needed to clarify the relationships.

Our results (cf. Table 4) clearly indicate that the control measures realized in the case study area were effective in reducing the number of large breeding sites: breeding site numbers dropped over time across all three building types. The effect was strongest for single-family house plots, followed by terraced building plots. For multi-family house plots, the effect of the control measurements per area was much lower, presumably because the number of breeding sites per hectare was very low already at the beginning of the breeding season. The other two breeding site categories seemed harder to control by the measures: for medium breeding sites, control measures were only effective for single family houses, while the other two building types showed an increase of medium breeding sites with time. For small sites no significant changes could be observed. A potential explanation for this mixed pattern could be that small and medium breeding sites need more constant attention—as, for example, watering cans are used regularly during summer months—while fixed large breeding sites need only little maintenance after an initial investment.

However, given the design of this study, we are missing a clear control group. While an controlled experimental design would have been beneficial from a scientific perspective, both the city administration which has ordered the control measures and the overwhelming majority of inhabitants would have objected to any experimental design which would have involved just counting breeding sites without associated control measures. The aim of this study was to investigate differences in the number of breeding sites and in *Aedes albopictus* abundance due to different building types, under conditions of an already established, area wide control program. Therefore, our findings need to be interpreted with care, and identified effects should be considered as associations and not as causal relationships. Future campaigns in new neighborhoods will try to incorporate a control group.

How well our results with respect to the effect of building type on breeding site density can be transferred to other suburban areas needs to be tested by replication studies. In general, we assume that trasferability is limited to areas with comparable building types and with an already established, area wide control programme. It seems interesting to investigate the importance of human behavior in more detail and to study how far different categories of human behavior—with relevance for *Aedes albopictus* control—can be linked to proxies, such as building typologies. The advantage of using indicators based on urban structure is that these can be easily computed for most regions based on available open data sources, such as OpenStreetMap.

### Factors influencing the egg count in the ovitraps

In addition to the abundance of breeding sites, the abundance of adult *Aedes albopictus* individuals is of main concern. Egg counts based on ovitraps are a frequently used indicator to estimate the abundance of *Aedes albopictus* due to the cost-efficiency of the approach [71]. However, mosquito monitoring only based on ovitrap counts has known shortcomings [71], e.g., due to the presence of alternative oviposition sites [72].

In our study area, the abundance of large breeding sites was negatively associated with maximum ovitrap egg counts (regression coefficient marginally significant, cf. Fig. 7). This could be linked to the larger variety of oviposition sites, making it less likely for female individuals to select an ovitrap as an ovipositioning site. For medium and small sized breeding sites no association was present in our data. This is in contrast with the expected behavior of a treehole nesting species which would be more likely to oviposition in smaller covered breeding sites. In a study by Yee et al. [73] *Aedes albopictus* have frequently ovipositioned in breeding containers up to 8.4 liters. The effect of the availability of large breeding sites might be due to oviposition skip, where females spread their eggs from a single gonotrophic cycle across multiple habitats—a behaviour that has been described for *Aedes albopictus* [74]. Fonseca et al. [75] have shown that female *Aedes albopictus* seemed to be less picky about oviposition site selection during summer months (as in our case study). Other studies have shown that large containers, such as water barrels or tubs, can host more larvae and pupae than smaller ones [10, 15]. This phenomenon may be due to the fact that large sites are more likely to collect food and are more resistant to desiccation [76]. We also hypothesize that biweekly egg counts—and, therefore, the biweekly replacement of the water—could further decrease the attractiveness of ovitraps as breeding sites. The pattern found in the data could be interpreted in the direction that fewer egg counts in ovitraps do not necessarily reflect a reduced *Aedes albopictus* population. In our case study, they might rather signal the availability/presence of more attractive artificial and possibly large breeding sites in the area. Consequently, our findings underscore the need for control measures to prioritize large breeding sites, as they may serve as population sustainers, most importantly—even in actively controlled areas.

When examining ovitrap counts, our results (cf. Fig. 7) showed that buffer areas with a greater share of plots with terraced houses tended to have larger egg counts on average. This effect was present after correcting for the association between terraced houses and abundance of large breeding sites. Therefore, this might indicate that terraced building plots should be given high priority with respect to mosquito control measurements. This could be done by visiting terraced building plots with a higher frequency or to spend more time on information during the visits here.

Uncontrolled areas, were negatively associated with egg-laying activity in ovitraps cf. Figs. 7 and 11). Our definition of "uncontrolled areas" has disadvantages, as it combines very different land-use types such inaccessible private properties, public roads and public green spaces. While these might differ in their ecological role, the availability of breeding sites could not be assessed. Therefore, we decided to classify these areas into one category. It could be, that some of the inhabitants that did not allow access to the building plot still control potential breeding sites. The legal framework offers no opportunity to asses a property for control measures without owner allowance. On public areas it would have been possible to assess the number of breeding sites if mandated by the city administration.

Although ovitrap egg counts declined in areas with greater proportion of uncontrolled plots, this does not necessaril imply lower *Aedes albopictus* populations. Rather, these plots may already hold conspecific larvae or pupae in hidden, unmanaged breeding sites, making them more attractive to gravid *Aedes albopictus* females than ovitraps [76–79]. As a result, these unseen breeding sites can sustain local *Aedes albopictus* populations despite apparently reduced egg counts in ovitraps. Such unmanaged sites might contribute to ongoing population stability, even in areas with active DtD measures in place done. Limitations in this predictor clearly lie in the undocumented number of breeding sites within uncontrolled areas. Depending on different factors such as vegetation, garden structure and owner interest of each individual plot of land, the effect of uncontrolled areas could vary strongly, yet it is still undocumented. With certainty, the displayed influence of uncontrolled areas in the present study emphasizes the importance of area-wide interventions across all plots of land in order to further achieve sustainable reduction of *Aedes albopictus* populations. A measure to control the uncontrolled sites is the sterile insect technique [e.g., 80] there sterilized males are released to reduce offspring numbers. The method had been successfully tested in Heidelberg [27] and applied in Baden–Württemberg in combination with the door-to-door control measurements as in our case study [30].

Several studies have mentioned the role of road drains in influencing ovitrap counts [81–85]. Depending on the type of the water drain they could potentially hold constant water and act as breeding sites [81–85]. In Pfaffengrund, road drains were controlled by Bti application by ICYBAC staff.

### Buffer size effects on egg count regression models

Our hypothesis of a 150-m radius for buffers around each ovitrap was based on mark–release–recapture (MRR) studies. Dispersal distances for *Aedes albopictus* indicated by these studies varied, however, influenced by the extent of urban plot of land density, green spaces or physical barriers [46, 49, 50] as well as by the blood feeding opportunities [15, 86, 87]. For instance *Aedes albopictus* in Heidelberg Ochsenkopf, were found to remain within 60–110 m of their release point [27, p.149]. Studies in India observed that some *Aedes albopictus* travelled a distance of maximum 100 m [49], while research in Italy reported travel distances of up to 200 m [50]. A study in Portugal reported mean distances traveled (MDT) between 58 and 161 m [47]. A MRR study in Albania reported MDTs between 77 and 99 m with a low number of individuals traveling between 200 and 250 m [48]. A Swiss research group documented longer dispersal distances beyond 250 m [46]. The regression models for the five buffer sizes suggest that the effects modeled were best captured at around 200 m within our study site. Explained deviance (c.f. Fig. 9) and confidence intervals of the regression coefficient estimates (c.f. Fig. 7) could be interpreted in this direction and would suggest a dispersal range leaning more toward 200 m. However, the regression models were only able to explain up to 29% of the deviance in the response, so unaccounted confounding factors—in addition to the high variability of the oviposition behavior and the underlying spatio-temporal population dynamics—might have influenced that result. In future work, we will test how far a finer distinction between the different plot types of uncontrolled areas might help to improve goodness of fit.

Transferability of the results with respect to the buffer size that best captured the relationships in the data to other regions is presumably limited to location of similar building structure and similar population dynamics of *Aedes albopictus*. Given the stable presence of *Aedes albopictus* in Pfaffengrund we assumed limited migration within the study area. Moreover, Pfaffengrunds suburban “garden city” layout [42, 44] with mainly inhabited plots of land and stable blood feeding opportunities likely does not require dispersal over longer distances for *Aedes albopictus*.

The effect of the buffer size on model performance could only be found for the GLMs fitted on the aggregated data. For the temporally disaggregated data the GLMM did not support the selection of any specific buffer size. This could be interpreted as a side effect of the strong quadratic temporal effect necessary to model the egg count over time. As the temporal development differed across the ovitraps, the hump shaped quadratic function fits not all ovitraps equally well. This effect might cover less important influences by other predictors. It was not possible to fit random slope models for a first- or second-order effect of *date* on the response in the model. Potentially, the variation in the temporal development could be captured by a generalized mixed additive model [53, 88]. Currently, we can only report partial support for the selection of a specific buffer size which might as well be due to an artifact of the data aggregation.

Despite the fact that ovitraps are indicative of the abundance of *Aedes albopictus*, it is important to note that the egg-laying activity may not accurately capture the exact proportion of the population during the study period [89, 90]. To gain a more comprehensive understanding of the *Aedes albopictus* populations and their dispersal, further methods such as BG Sentinel traps [91], human landing or larval sampling could be integrated into future studies [92].

## Conclusions

Our results indicate, that it is possible to relate building type with both the distribution of breeding sites and ovitrap egg counts of *Aedes albopictus*. Especially terraced building plots seem to be hosting more breeding sites per area, while multi-family housing plots showed a much lower breeding site density. This might help to prioritize control measurements across cities. Our results further showed, that the control measures in place were effective with regard to the decreasing the number of large breeding sites. The number of medium and small breeding sites did not respond to the control measures—with the exception of medium breeding sites at single-family houses. Further research is needed to better understand how human behavior shape the abundance and size distribution of breeding sites and to test tailored interventions to reduce breeding sites even more effectively. With respect to the oviposition pattern in ovitraps, our results underline the complexity of estimating *Aedes albopictus* density simply based on ovitrap egg count data. Higher egg counts might simply indicate the unavailability of alternative oviposition sites. The positive association between the share of terraced buildings and the ovitrap egg count provides additional support for the hypothesis, that consideration of building typologies and urban structure might help to prioritize control measures.

## Data Availability

Due to data privacy regulations the data cannot be made completely publicly available. The preprocessed data without spatial coordinates along with the RMarkdown code used to generate the results is available at https://github.com/slautenb/effectOfBuildingTypesOnAedesAlbopictusInHeidelberg.
